# Anticancer Activity and Underlying Mechanism of Phytochemicals against Multiple Myeloma

**DOI:** 10.3390/ijms20092302

**Published:** 2019-05-09

**Authors:** Beomku Kang, Hyunmin Park, Bonglee Kim

**Affiliations:** 1 College of Korean Medicine, Kyung Hee University, Seoul 02453, Korea; beomkukang@khu.ac.kr; 2 Department of Pathology, College of Korean Medicine, Graduate School, Kyung Hee University, Seoul 02453, Korea; rapha837@khu.ac.kr

**Keywords:** multiple myeloma, phytochemicals, natural products, anticancer, apoptosis, cell cycle, angiogenesis, miRNA, clinical trials

## Abstract

Multiple myeloma (MM)—a common hematologic malignancy of plasma cells—accounts for substantial mortality and morbidity rates. Due to the advent of novel therapies such as immunomodulatory drugs (IMiDs), proteasome inhibitors (PIs), and monoclonal antibodies (mAbs), response rates were increased and free survival and overall survival have been elevated. However, adverse events including toxicity, neuropathy or continuous relapse are still problems. Thus, development of novel drugs which have less side effects and more effective is needed. This review aims to recapitulate the pharmacologic anti-MM mechanisms of various phytochemicals, elucidating their molecular targets. Keywords related to MM and natural products were searched in PUBMED/MEDLINE. Phytochemicals have been reported to display a variety of anti-MM activities, including apoptosis, cell cycle arrest, antiangiogenesis, and miRNA modulation. Some phytochemicals sensitize the conventional therapies such as dexamethasone. Also, there are clinical trials with phytochemicals such as agaricus, curcumin, and Neovastat regarding MM treatment. Taken together, this review elucidated and categorized the evidences that natural products and their bioactive compounds could be potent drugs in treating MM.

## 1. Introduction

Multiple myeloma (MM) is the neoplasm of plasma cells, accounting for up to 10% of hematologic neoplasm [1]. The current status of MM is dreadful: the median survival period of patients with stage III MM is approximately 29 months, while patients with stage II and stage I MM live for approximately 44 and 62 months, respectively [2].

### 1.1. Current Drug Therapies for MM—And Their Limitations

As the incidence and mortality of MM increases, various novel therapies have been developed, such as target drug therapies (IMiDs, PIs, mAbs, etc.), combination therapies (bortezomib-based, lenalidomide-based, etc.), corticosteroids, stem cell transplantation (autologous allogenic), radiotherapy, and bisphosphonate treatment. The most recent advances of the novel targeted drug therapies are shortly presented below. Immunomodulatory drugs (IMiDs) induce apoptosis by regulation of the immune system, via modulation of T cell activity and cytokine production, such as tumor necrosis factor (TNF)-α and interleukin (IL)-1β and IL-6 [3,4]. Thalidomide, the first in the development of IMiDs, was introduced in 1999 and has undergone various in vitro and clinical phase II studies, which evaluate both the independent and combined efficacy of thalidomide with other drugs, such as dexamethasone [5,6,7,8,9,10,11]. However, thalidomide is considered to have severe side effects including dizziness, drowsiness, constipation, and muscle weakness [12]. Proteasomes are usually responsible for maintaining cellular homeostasis by controlling regulatory proteins [13]. The first generation of proteasome inhibitors (PIs) includes bortezomib, which has undergone various phase I–III studies over the past decades [14,15,16]. However, bortezomib has been reported with grade III adverse effects such as thrombocytopenia, fatigue, peripheral neuropathy, etc. [17]. Monoclonal antibodies are new protein therapies, once called ‘magic bullets’, whose mechanism mainly centers on blocking ligand binding/signaling to alter growth rates or to induce apoptosis [18]. The most well-known medication for MM, daratumumab, is a human CD38 monoclonal antibody that engenders cellular toxicity in MM cells [19]. However, severe side effects of licensed monoclonal antibodies have been reported, such as immune reactions (acute anaphylaxis, serum sickness, etc.), cardiotoxicity, and cytokine release syndrome (CRS) [20,21,22,23,24,25,26].

### 1.2. Phytochemicals: A Possible Solution in Overcoming the Limitations of Contemporary Therapies?

Although the novel therapies extended the expected life span of MM patients, there are concerns about the side effects with prolonged use of the medications. As researchers realized the impending drawbacks of current chemotherapies, they focused on natural products to replace the current therapies. Natural products have benefits in that they (i) display metabolite-likeness, (ii) are active transport metabolites, and (iii) express high levels of bioavailability [27,28,29]. Due to these factors, studies that report the potent activities of natural products have skyrocketed in the last 30 years [30]. For instance, Raimondi et al. reviewed around thirty natural compounds treating MM including natural compounds possessed analgesic activity for clinical translation purpose [31]. In this study, natural products, reported as their anti-MM effects, are collected and reviewed by their mechanisms—apoptosis, cell cycle arrest, antiangiogenesis, and miRNA regulation—and results of clinical trials to evaluate the potential of future development as an anticancer agent.

## 2. Phytochemicals and MM

### 2.1. Anti-MM Effects of Natural Products via Intrinsic/Extrinsic Pathways of Apoptosis

**Figure 1:**
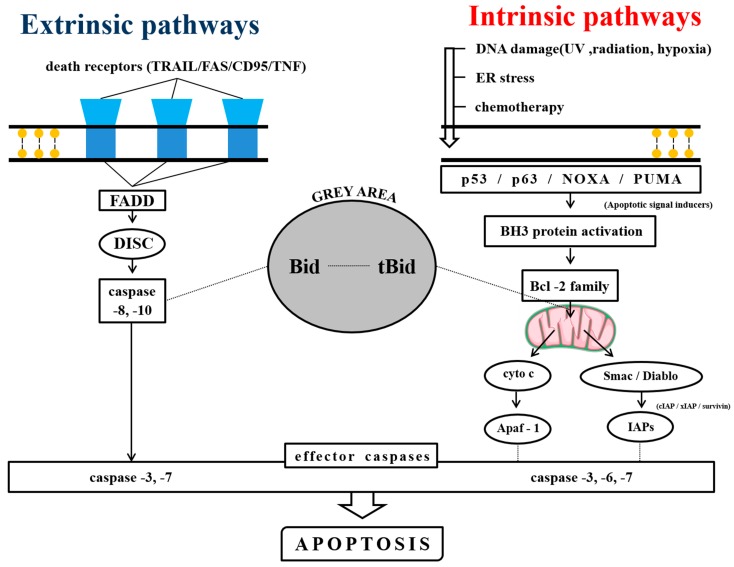
The extrinsic and the intrinsic pathways of apoptosis. The left side shows the extrinsic pathway and its mediating factors. The right side shows the intrinsic pathway and its activating mechanism. The grey area in the middle shows the controversial area of Bid proteins.

Apoptosis is a physiological process to maintain the homeostasis of cells [32], and contemporary pharmaceutical research mainly centers on using its various mechanisms of action to design anticancer drugs. There are mainly two that regulate apoptosis: the extrinsic and the intrinsic pathways [32] (Figure 1).

**Figure 2:**
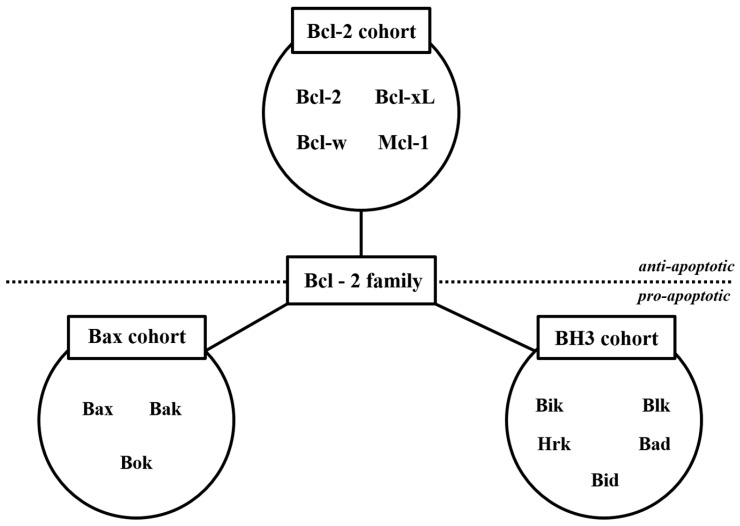
The Bcl-2 family proteins. The Bcl-2 family proteins play crucial roles in the apoptosis process. The upper side shows antiapoptotic factors, while the lower side shows proapoptotic factors.

The intrinsic pathways are mainly controlled by the proteins of Bcl-2 family (Figure 2). There are mainly two subcategories; antiapoptotic proteins (Bcl-2 subfamily cohort) and proapoptotic proteins (Bax subfamily cohort and BH3 subfamily cohort) [33].

The anti-MM natural products were categorized by their mediating pathways: (i) the intrinsic pathway (Table 1), (ii) the extrinsic pathway (Table 2), and (iii) intrinsic and extrinsic pathways (Table 3).

#### 2.1.1. Natural Products Induce Intrinsic Apoptosis

Among the various natural products that induce intrinsic apoptosis is the chloroform fraction (CHCl3) of *Azorella glabra* Wedd., containing polyphenols, flavonoids, terpenoids; induced apoptosis via caspase-3 activation; cleavage of PARP; and repression of Bcl-2 in RPMI8226, SKMM1, MM1S, and MM cell lines [34]. Berberine, a natural isoquinoline alkaloid that is mainly extracted from *Coptis chinensis* Franch., increased ROS generation and displayed potent apoptotic activity. Berberine significantly downregulated miR-21, which targets Bcl-2 family proteins, thus decreasing the expression of Bcl-2 and increasing PUMA and cleaved (c)-caspase-3, -9 expression [35]. Brazilin, derived from *Caesalpinia sappan* (L) Todd., inhibited histone deacetylases (HDACs), which are enzymes that control histone acetyltransferases (HATs) [36]. Cleavage of caspase-3 and PARP increased, and the expression levels of Bcl-xL and Bcl-2 were repressed, but the level of Mcl-1 remained unchanged by brazilin treatment [37]. Boswellic Acid (AKBA), derived from *Boswellia serrata* Spreng., displayed a time-dependent activation of c-caspase-3, inhibiting expression of survivin, Bcl-xL, Bcl-2, and Mcl-1, with the maximum suppression observed at ~12–24 h. Also, cleavage of PARP protein was examined, suggesting a caspase-3 dependent apoptosis [38]. CSTMP, a newly designed and synthesized TMP (tetramethylpyrazine, extracted from *Ligusticum wallichii* Franch.), and resveratrol derivative, increased the mRNA level of Bax, and decreased the mRNA level of Bcl-2, Bcl-xL, and activated caspase-3, -8, -9. Also, CSTMP increased ER stress related proteins (CHOP, c-caspase-12, GRP78, GRP94) after 48 h, upregulating the expression of PERK, eIF2α, IRE1α and ATF6 (ER stress related key signals) [39]. Curcumin, from *curcuma longa* Linn, activated caspase-3 and -8, released cytochrome C (cyto C), and also cleaved Bid [40]. Emodin, from *Rheum palmatum L.*, induced apoptosis through inhibition of JAK2/STAT3/Mcl-1 pathway. Emodin inhibited IL-6-induced JAK2 kinase activity, resulting in diminished STAT3 activity and a decrease in Mcl-1 expression. Also, pro-caspase-3 and -9 expression levels decreased, but caspase-8 was not cleaved [41]. Genipin, an active compound derived from *Gardenia jasminoides* J. Ellis, suppressed STAT3 activity by repressing c-Src and also downregulating the target genes of STAT3, including Bcl-2, Bcl-xL, survivin, cyclin D1, and VEGF. Also, genipin exhibited synergistic effect with other chemotherapeutic agenst such as bortezomib, thalidomide, and paclitaxel [42]. Compound K (CK), from *Panax Ginseng* C.A.Mey., induced apoptosis via STAT3 pathway, decreasing levels of Bcl-xL, Bcl-2, and survivin, as well as cleaving PARP and caspase-3 [43]. Matrine, a main alkaloid of *Sophora flavescens* Aiton, was examined with the loss of mitochondrial membrane potential (MMP or Δψm), inducing cyto C release from mitochondria to cytosol, accompanying decrease of Bcl-2 and increase of Bax, resulting in caspase-3 activation [44]. Oridonin, a natural diterpenoid extracted from *Rabdosia rubescens* (Hemsl.) H.Hara, mainly decreased expression of Mcl-1 and Bcl-xL, but the Bcl-2 level was unchanged [45]. Pomegrante extract, derived from *Punica granatum* L., inhibited MMP, thus inducing apoptosis [46]. Resveratrol, from *Veratrum grandiflorum* L., showed downregulation of cyclin D1, cIAP-2, XIAP, surviving, Bcl-2, and Bcl-xL, as well as increase in Bax. Resveratrol also exhibited decrease in Bfl-1/A1, and TRAF2, which are controlled by NF-κB, inducing downregulation of Akt [47]. *Scutellaria baicalensis* Geogi (SB) extract elevated expression of cyclin-dependent kinase inhibitors (CDKIs) p27^KIP1^, but CDKI p21^WAF1^ was unchanged. Also, mitochondrial injury was observed, along with a decrease in p53 level, leading to an increase in Bax and decrease in Bcl-2 and Bcl-xL [48]. SN extract, derived from *Strychnos nux-vomica* L., suppressed the MMP, leading to release of cyto C into the cytosol, thus inducing apoptosis [49].

#### 2.1.2. Phytochemicals Mediated through Extrinsic Pathways

Thymoquinone (TQ)—a phytochemical compound found in *Nigella sativa* Linn—increased the surface expression level of CD95 exceptionally, while decreasing the cytoplasmic CD95 expression indicating that TQ displays its effect by relocating the intracellular CD95 to the surface of the cell without any de novo CD95 protein synthesis [50].

#### 2.1.3. Phytochemicals Mediated through Both Intrinsic and Extrinsic Pathways

Alipidin—a cyclic depsipeptide extracted from *Aplidium albicans*—induced apoptosis by regulation of the intrinsic pathway and exhibited loss in the MMP, which leads to a decrease in Mcl-1 level. Mediated by the extrinsic pathway, upregulation of GADD45A, GADD45B, TRAIL, CASP9, CASP6, CIDEC were examined, along with the cleavages of PARP, caspase-3, -7, -8, and -9, and Fas/CD95 translocation into lipid rafts [51]. Cantharidin, a derivative of *Blister beetles*, exhibited three main effects: (1) loss of MMP and the activation of caspase -3 and -9 were mediated via the intrinsic pathway, (2) increase of Fas and cleavage of Bid protein were mediated by the extrinsic pathway; and (3) downregulation of Bcl-xL was due to modification of STAT3 pathway [52]. Also, dolastatin 15, a peptide derived from *Dolabella auricularia*, induced loss in MMP, which activated caspase -3, -9, and -8. In addition, cleavage of Bid protein and the activation of Bax protein were observed. Furthermore, dolastatin induced apoptosis via Fas(CD95)/Fas-L(CD95-L) pathway [53]. Epigallocatechin-3-gallate (EGCG), a polyphenol extracted from *Camellia sinensis* (L.) Kuntze, activated the p63 protein (p53-like protein involved in apoptosis), and also the elevated expression of death-associated protein kinase 2 (DAPK2), Fas, Fas ligand, and caspase-4 was observed [54].

### 2.2. Anti-MM Effects of Natural Products via Cell Cycle Arrest

The mammalian cell cycle consists of mainly four stages—G1, S, G2, and M phases—and exceptionally, the G0 phase, for resting, nonproliferating cells in our body. Cell cycle arrest is mainly induced by checkpoints that exist in various points of the cell cycle. Cell cycle arrest and apoptosis are closely related, since cells unable to be fully repaired usually accompany programmed cell death [55].

**Figure 3:**
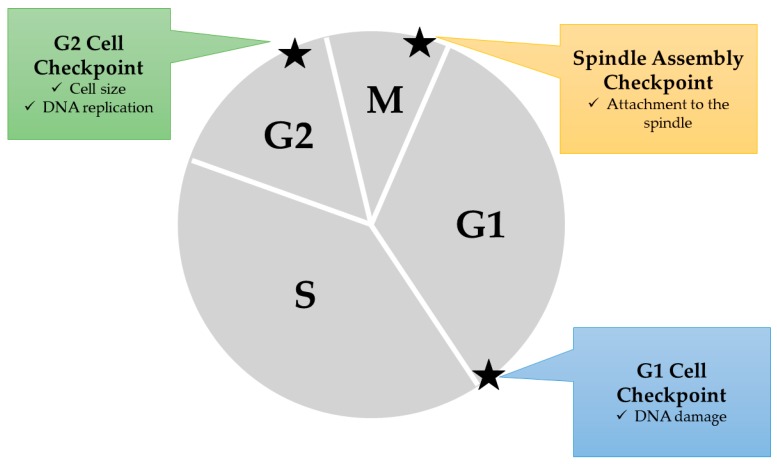
Cell cycle checkpoints inducing cell cycle arrest. Three points in the cell cycle are usually determined for normal cell growth. At the G1 cell checkpoint, when DNA damage is observed, the cell automatically turns to the G0 state (resting state), thus stopping the cell from growing any further. At the G2 cell checkpoint, the cell size is observed and the DNA replication is examined. When either of these processes is not yet complete or erroneous, cell cycle arrest is induced. At the spindle assembly checkpoint, whether the chromosomes are attached to the spindle is examined.

In cell cycle arrest, checkpoints play a crucial role in guaranteeing that the cell has completed the necessary procedures for each phase (Figure 3). There are mainly four checkpoints in the cell cycle that regulate the whole cycle. First, the G1/S checkpoint blocks cells whose DNA has been damaged. Second, the intra-S phase checkpoint blocks cells that have been impaired by genotoxic stress, but if delayed, the delay is only transient, compared to other checkpoints. Thirdly, the G2/M phase checkpoint blocks entrance into M phase if DNA replication is incomplete. Finally, the spindle checkpoint blocks entrance into anaphase of the M phase if the chromatids are not aligned on the mitotic spindle [56,57,58,59].

There are complex agents regulating the pathways of cell cycle arrest checkpoints, but in this review, we focused on checkpoints which were inhibited by the natural products. Natural product-derived drugs exhibited accumulation of cell population in various stages of the cell cycle, thus causing cell death (Table 4). Aplidin, from *Aplidium albicans*, exhibits an increase in the percentage of cells in the G2-M phase. Also, evident sub-G0 accumulation was observed in cells treated with higher doses of Aplidin. This suggests that Aplidin blocks the proliferation of MM cells as well as induces cell dead [51]. Dolastatin 15, a peptide derived from *Dolabella auricularia*, expressed a decrease of cells in the G1 phase and a following accumulation of cells in the G2/M phase. Also, increase in hypodiploid cells were observed, indicating dolastatin exhibits G2/M cell cycle arrest accompanying apoptosis [53]. Triptolide, derived from *Tripterygium wilfordii* Hook. f., exhibited increase in G0/G1 phase cells, as well as a decrease in S phase, while the cells in G2/M phase remained constant. Also, it has been observed that higher concentrations could exhibit G0/G1 phase cell accumulation at shorter periods [60]. Brazilin, isolated from *Caesalpinia sappan* (L.) Tod., exhibits cell cycle arrest at the G2/M phase. Pomegrante extract, derived from *Punica granatum* L., induces G2/M and S phases cell cycle arrest. The leave extracts induced 59% cell cycle arrest in G2/M phase, but the S phase cell population remained unchanged. The flower extract induced 62% cell cycle arrest in G2/M phase, accompanying 8.6% cell cycle arrest in S phase [46].

### 2.3. Anti-MM Effects of Natural Products via Antiangiogenesis

**Figure 4:**
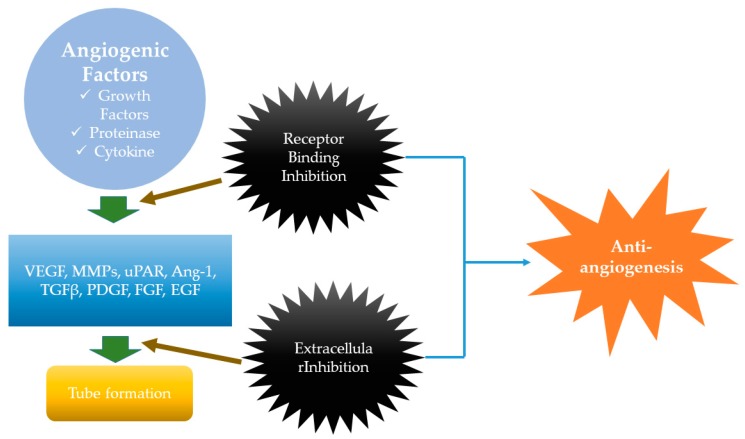
Angiogenesis mechanisms. Angiogenic growth factors induce the angiogenesis in tumor cells. This mechanism is usually mediated by factors such as VEGF (vascular endothelial growth factor), MMPs (matrix metalloproteinase), uPAR (urokinase receptor), Ang-1 (angiopoietin-1), TGFβ (transforming growth factor β), PDGF (platelet-derived growth factor), FGF (fibroblast growth factor), and EGF (epidermal growth factor). This leads to tube formation. However, during these processes, various inhibitions occur, such as receptor binding inhibition and extracellular inhibition. These lead to inhibition of angiogenesis, or, as mentioned in this review, “antiangiogenesis”.

Angiogenesis has been well-known as the indicator of tumorigenesis, in that new blood vessels form during the process, ensuring that the tumor receives enough blood supply [61]. The most current review on the mechanisms of angiogenesis state that there are largely two regulators related to angiogenic activity. Positive regulators of angiogenesis refer to factors that promote angiogenesis, such as vascular endothelial growth factor (VEGF), platelet-derived growth factor (PDGF), fibroblast growth factor (FGF), epidermal growth factor (EGF), transforming growth factor β (TGFβ), matrix metalloproteinases (MMPs), TNF, angiopoietins (Ang-1), and urokinase receptor (uPAR) (Figure 4). Especially, VEGF has significant importance because it promotes angiogenesis in various tumors, inflammation, and healing in wounds. VEGF has various expression regulators, such as hypoxia and cytokines [62]. There are negative regulators of angiogenesis—often called angiogenesis inhibitors—which are either endogenous or synthetic. Examples for endogenous angiogenesis inhibitors include interferon, interleukins, and tissue inhibitors of MMPs, angiostatin, and endostatin. Synthetic angiogenesis inhibitors refer to angiogenesis carried out by drugs [63].

Angiogenesis in hematological malignancies were once thought as only contributing trivially in the tumorigenesis, but recent research has proved otherwise. The bone marrow (BM) vasculature has been commonly observed in hematological cancer patients. Therefore, the vasculature of BM is considered as a major indicator in detecting various hematological disorders, in that an increase in BM vasculature density usually indicates induction of angiogenesis [64]. Although understanding the molecular mechanisms behind angiogenesis in “liquid tumors” are being thoroughly researched, natural products that exhibit anti-MM activity by inhibiting angiogenic parameters were researched (Table 5).

Bruceantin, derived from *Brucea javanica*, was examined to reduce all angiogenic parameters, including total branch length, total segment length, number of anchorage junctions, branches, segments, junctions, and nodes. The significant changes were induced at 12.5–50 nM [65]. Wogonin, an active monoflavone in *Scutellaria baicalensis* Georgi, was used in both in vitro and in vivo experimental settings. In in vitro experiments both protein and mRNA levels of VEGF expression were reduced. Furthermore, c-Myc and HIF-1a expression at protein levels were reduced. In in vivo experiments, expression levels of c-Myc, HIF-1a, VHL, and VEGF decreased, were observed in mice. Wogonin exhibited anti-MM activity without damaging the physiological functions of vital organs. Furthermore, combination therapy with bortezomib and lenalidomide induced further repression of MM [66]. Bergamottin, a natural furanocoumarin derived from grapefruit, exhibits potent antiangiogenic properties downregulating the levels of gene products regulated by STAT3, for example, COX-2, VEGF, cyclin D1, survivin, IAP-1, BCL-2, and Bcl-xL [67]. Solenopsin A, a primary alkaloid from the fire ant *Solenopsis invicta* Buren, showed antiangiogenic effects in in vitro and in vivo settings. Given that solenopsin A displayed antiangiogenic activities in in vitro experiments, the proliferation assay showed that solenopsin A inhibited cell proliferation and downregulated Akt levels. Since Akt is related to apoptosis and angiogenesis, this leads to further examination in in vivo experiments. In vivo studies showed that solenopsin A inhibits embryonic angiogenesis in zebrafish models. Distinct from other angiogenesis examinations, the vasculogenic vessels (dorsal aorta and posterior cardinal vein) formed appropriately in solenopsin-treated zebrafish embryos. This suggests that solenopsin may delay angiogenic precursors or sprouts from reaching their target [68]. Rutin–zinc (II) flavonoid complex, derived from *Carpobrotus edulis* (L.) N.E.Br., decreases VEGF and cyclin D1 expression levels, while upregulates the gene expression levels of caspase -3 and -8 [69]. Artesunate, a sesquiterpene lactone isolated from *Artemisia annua* Linn, exhibits angioenic activities in both in vitro and in vivo assays. When treated with artesunate, the number of vascular endothelial cells decreased, along with HUVEC migration. In in vitro assays, the density of vascular sprouts decreased, which was parallel to the decrease of the average number of vessels examined in in vivo assays [70].

### 2.4. Anti-MM Effects of Natural Products via miRNA Regulation

MiRNAs, the short RNA molecules that bind to mRNA, are reported to regulate one-third of the gene in human genes. Various biological activities such as metabolism, differentiation, and growth are related to miRNA functions and many of them have been reported to play certain roles in many human diseases including cancer [71]. We collected two categories of miRNAs that correlate to the two pathways we have dealt in this review, with the help of the most updated review available on the subject of miRNA and cellular mechanisms [72]. Firstly, about the IL-6/STAT3 pathway, upregulation of miR-21 initiates the activation of IL-6, JAK and STAT3 pathways, which in turn triggers the antiapoptotic genes Bcl-xL, Mcl-1, and c-Myc [73,74]. The deviation of miR-21 has been thought to be a major indicator of an early onset of MM [75]. Also, the overexpression of miR-20a has shown to downregulate apoptotic genes BIM and SOCS-1, which has been examined to be a negative regulator of the IL-6/STAT3 pathway [76].

Secondly, the miRNAs related to the p53 gene-related pathway have been regarded as potential therapeutic targets in MM, because they correlate with the p53 pathway [77]. The downregulation of p53 has been observed to decrease the cell cycle arrest related proteins (p21, GADD45A, etc.), apoptosis-related proteins (Bax, PUMA, etc.), while promoting angiogenesis (TSP1, maspin) [78,79]. MiR-196b induced cell cycle arrest at G1/S phase [80] and miR-215 directly repressed the transcriptional target of p53 [77,81]. Furthermore, miR-150-5p has been a potential target of surviving [82], and also reported to induce VEGF production and tumor growth via angiogenesis [83].

The direct correlation between miRNAs and MM was best observed with relations to the bone marrow microenvironment (BMM) [84]. Various researches focused on determining the levels of miRNAs in MM settings. MiR-29b was examined to reduce growth and induce apoptosis in MM cells, targeting proteins such as Mcl-1 [77,85]. MiR-34a is examined to target Bcl-2, inducing growth inhibition and apoptosis in MM cells [86]. Also, miR-92a and miR-125a-5p targets VEGF and p53, respectively [87,88]. MiRNAs have been playing significant roles in defining the mechanisms behind tumor progression and continuing researches on the relationship between certain miRNAs and MM. Precedent researches have been reported about natural products that have shown to control miRNA levels, inducing anti-MM activity (Table 6).

Berberine, a natural alkaloid derived from *Coptis chinesis*, downregulates miR-21 levels via IL-6/STAT3 pathways and upregulates the expression of programmed cell death 4 (PCDC4), which in turn suppresses the p53 pathway [35,89]. Another mechanism of berberine is by inhibiting the NF-κB nuclear translocation, through the Set9-mediated lysine methylation. This results in reduced level of miR-21 and Bcl-2, which leads to ROS generation and apoptosis [35]. Moreover, berberine inhibits proliferation of MM cells by decreasing levels of miR-17-92, miR-99a-125b, and miR-106-25 clusters, etc. which are known as onco-miR in MM [90]. Triptolide, derived from *Tripterygium wilfordii* Hook. F., significantly decreases levels of miR-142-5p and miR181a, effectively inhibiting glucocorticoid receptors (GR). Also, effective combination has been proved of triptolide with combination of dexamethasone [91].

Although current research for novel targets that aim to target levels of miRNA expression is being carried out widely, in vitro and in vivo experiments regarding the effects of natural product-derived drugs on miRNA expression is scarce. Out of all natural product research, miRNA research should be invigorated.

### 2.5. Clinical Trials of Natural Products on MM

Clinical trials pose a significant value in testing a potential anticancer drug in that it gives the final confirmation to apply it to clinical settings. We have thoroughly researched every clinical trial that were listed on www.ClinicalTrials.gov and came across the studies, as mentioned below (Table 7).

The first clinical trial (clinical trial number: NCT00970021), a completed study with the purpose of researching the clinical properties of *Agaricus blazei* Murrill (ABM) (a mushroom extract shown to exhibit potent antimyeloma activity in mouse models [93]) was established to examine the effects of ABM as a supplementary treatment in addition to normal chemotherapy. Quadruple masking was done to participants, care providers, investigators, and outcome assessors. Excluding those who withdrew from the trial, a total of 33 patients (16 in the ABM treatment group and 17 in the placebo group) were enrolled in the study, in all sexes. The intervention was implemented in the following method; patients were randomly divided into two groups and each group was prescribed 60 mL of agaricus extract or placebo (depending on groups) once daily from the start of stem cell mobilizing therapy until one week after the end of aplasia after chemotherapy, a high dose of melphalan. The mean overall survival was 50.7 months in the agaricus group and 47.4 months in the placebo group. The outcome was evaluated mainly by the cytokine levels in serum. Treated with ABM, IL-1ra, IL-5, and IL-7 serum levels were significantly increased. The proinflammatory chemokine MCP-1 was downregulated in the ABM treated group. But a major difficulty in examining the results in this study arose because of the composition of ABM and its estimated mechanism is unclear. The polysaccharides phytocomplex is thought to help exhibit its immunomodulating and anticancer properties, and although clinical studies have been positive in its results, there has been concerns for agaritine (estimated to be contained in ABM), which is a well-known carcinogenic and toxic substances. Clinical trials regarding the potential toxicity of ABM should be initiated in order to fully examine the clinical properties of ABM [92,94].

The second completed study (clinical trial number: NCT00113841) tested the effect of curcumin with or without bioperine in patients with MM. The main objectives were (i) to evaluate whether curcumin exhibited anti-MM activity alone or with bioperine, (ii) investigate the pharmacological parameters of curcumin and determine the effect of bioperine to curcumin in combination treatments, and (iii) the change in NF-κB protein levels and related genes in patients treated with curcumin alone or with bioperine. This trial was an open label, randomized pilot study. A total of 33 patients (nine patients were excluded due to screening failure) were enrolled in this study (curcumin-treated patients: 16; curcumin with bioperine treated patients: 17), and both sexes were included. The study starts with six patients in total, three patients in each arm (curcumin alone or with bioperine). After enrollment of each six patients, a new patient group will be included, with a higher dose at every level. The baseline dose starts with 2 g of curcumin (5 g of bioperine) per day. Evaluation and physical examination were conducted every four weeks. The outcomes were measured in the percent change of NF-κB protein expression in peripheral blood mononuclear cells (PBMCs) from baseline through four weeks of treatment. The outcome results were evaluated in both curcumin alone group and curcumin with bioperine group. The former group showed a decrease in the NF-κB protein expression in PBMCs by 21%, while the latter group showed a decrease by 37% (*p* = 0.16). This suggests that drug treatment using curcumin with bioperine has some significance in treating MM via the regulation of NF-κB.

The third completed study (clinical trial number: NCT00022282) investigated the effect of AE-941, which is shark cartilage extract more often known as the name Neovastat, on patients with relapsed or refractory MM. This was a phase II, multicentered, single-arm, open-label study with the objective of (i) testing the safety of AE-941 and (ii) determining/checking the duration of tumor response rates. A total of 125 patients were enrolled in this study, and AE-941 was prescribed twice a day, 240 mL per day. The primary completion date was March 2007, but no further study results were updated [95].

The three clinical trials show that natural products have ample significance in becoming potential therapeutic drugs in clinical settings. Although the cases are few, it has shown meaningful results, urging the need to carry out more trials in the future. But since the drugs mentioned above were studied mainly into phase II studies, further studies should be carried out to reconfirm the safety and maximum efficacy.

## 3. Discussion

Natural products have a long history in human’s quest for cure the diseases, and for the past few decades, researcher’s interest has spiked due to the limitations of conventional therapies and the effectiveness of natural product-derived drugs [96]. These current trends are especially important in combating cancer in general, and specifically MM, because of its dreadfulness. In fact, our team consistently reported the anti-MM natural products such as brazilin [37], β-sitosterol [97], *Salvia miltiorrhiza* ethanol extract [98], and *Cnidium officinale* Makino ethanol extract [99]. The incidence of patients with newly diagnosed MM is approximately 14,000 cases each year, only in the United States and the causes of MM still remain mainly unknown [100]. Not only newly diagnosed MM, but also relapsed or refractory MM is also a problem, since MM might recur in the long-term. In the face of these setbacks, and in line with the current trend, phytochemicals are surely a promising area for researchers. Thus, this review has mainly focused on the basic therapeutic mechanisms of phytochemicals in MM. Phytochemicals showed anti-MM activities by induction of apoptosis, cell cycle arrest, inhibition of angiogenesis, the modulation of miRNAs, sensitizing the conventional therapies [101].

During the course of this review, we collected data of previous studies and analyzed the efficacies of the drugs. First of all, we focused on the dosage of the drug used in each experiment. In researching the apoptotic mechanism of natural products, experiments that used dosages higher than 100 μmol/L should have had a control group to guarantee the safety in normal cell lines. However, normal cell lines were not tested in any of the experiments that used unexceptionally high dosages.

Also, experiments that researched the antiangiogenic effects of natural products should have tested the cytotoxicity of the drugs on cancer cell lines to make sure that the antiangiogenic effects were not induced by the cytotoxic effects of natural products. Antiangiogenic effect refers to when cancer cells lack in the proliferation of blood vessels via oxygen depletion etc. However, without cytotoxicity assays, it cannot be confirmed whether the effects are due to angiogenic effects or cytotoxic effects.

Nonetheless, experiments that coupled in vitro and in vivo experiments are can be appraised highly because most experiments were conducted only in in vitro [64,66]. To determine the efficacy and safety of drugs not only on cell lines but also in animal models, in vivo experiments are essential. However, there seems to be a number of researches that has insufficient data reliable conclusions. For example, studies that used extracts as test drugs should have done analysis on the components of the extract by methods such as HPLC, since extracts tend to vary in its constituents by the way drugs are sampled. Only the paper on SN root extract determined the chemical finger print of the used drug using LC-mass spectral analysis [47]. Verifying the components of drugs used in the experiment is crucial because quantification seems to be a major challenge in natural product-derived drug research.

Recent studies demonstrated that epigenetic modifications play significant role in cancer initiation and progression which include noncoding RNA methylation [102]. Short and long noncoding RNAs have been reported their effects in cancers, including MM. Amodio et al. revealed the biological role and therapeutic effect of long noncoding RNA (lncRNA) metastasis-associated lung adenocarcinoma transcript 1 (MALAT1) in non-small cell lung cancer, breast cancer, hepatocellular carcinoma, ovarian cancer, cervical cancer, lymphoma, and MM cell lines [103,104]. In addition, oncogenic microRNAs (miRNAs) including miR-21 and miR-17-92 have been reported their potential of therapeutic target in MM therapies [105,106].

In this review, we have only dealt with natural products that display potent anti-MM effects. Nonetheless, caution should be needed because not all natural products are safe. There are some natural products that showed side effects in experimental settings. For example, vinca alkaloids, derived from the pink periwinkle plant *Catharanthus roseus* G. Don, display hypoglycemic and cytotoxic effects but have dose-limiting toxicity such as neutropenia [107]. Also, anthracycline, derived from *Streptomyces peucetius* was reported to show cardiotoxicity in patients [108]. Taking into view these facts, clinicians should take caution in clinical trials testing natural products. Presently, the data accumulated from in vitro studies offer a generous platform for further research on natural compounds as potential therapeutic targets in MM. More advanced studies in animal models are essential to valorize the currently available information on the mechanism of action. The scarcity of in vivo and clinical studies, coupled with the positive effects documented by in vitro investigation, represent a strong incentive to continue a meaningful work in this area of cancer research.

## 4. Materials and Methods

**Figure 5:**
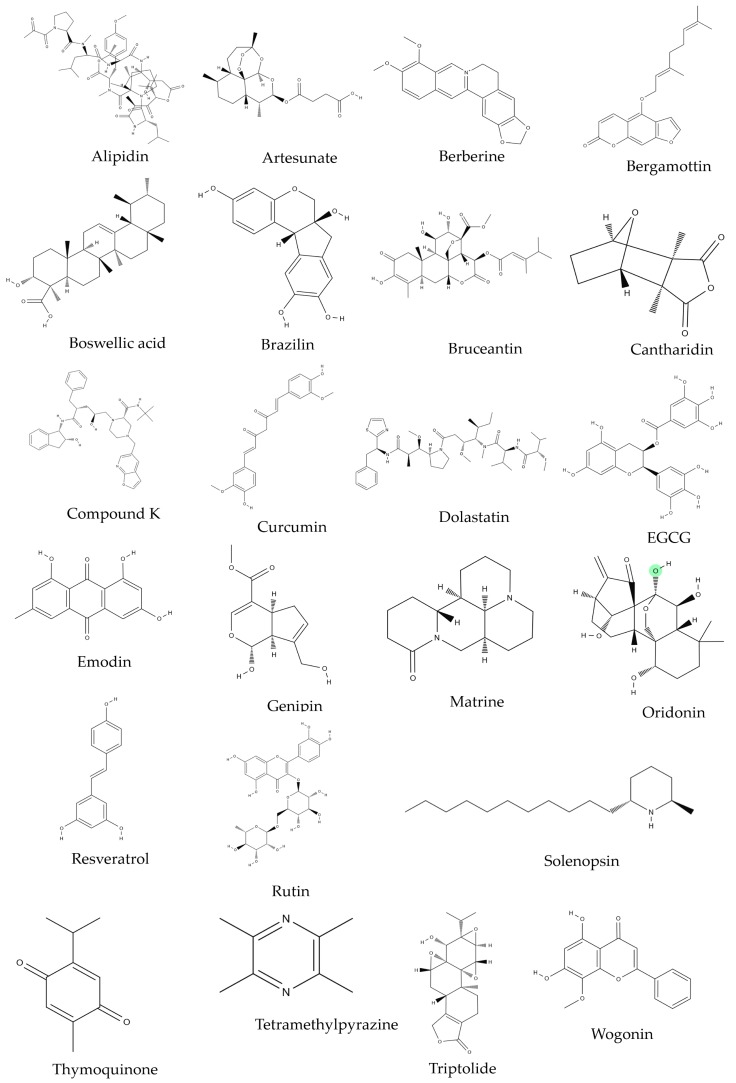
The chemical structures of phytochemicals which demonstrated anti-MM efficacies in vitro.

Studies regarding the effect of natural product on MM were collected from PUBMED/MEDLINE (www.ncbi.nlm.gov/pubmed) and Google Scholar (http://scholar.google.com). The keywords “multiple myeloma and natural products”, “multiple myeloma and herbs”, and “multiple myeloma and marine products” were used. Criteria:(1) researches based on cell line/clinical trials that were done on human MM cell lines or human multiple myeloma patients, (2) researches that had reliable statistical analysis data (*p*-values that is less than 0.05), and (3) researches that were not upset by subsequent case reports or experiments. The collected data was then classified into five main categories by their mechanisms of action and their clinical application: (i) apoptosis, (ii) cell cycle arrest, (iii) antiangiogenesis, (iv) microRNA regulation, and (v) clinical trials. This review focused on the basic physiological or pathological mechanisms of each category. The family names of natural products were imported from a reliable source (http://www.theplantlist.org/). Natural product-derived compounds and the chemical structures mentioned in MM research were double-checked from the NCBI PubChem website (http://www.ncbi.nlm.gov/pccompound) for precise information. The chemical structures of compounds were illustrated in Figure 5.

## 5. Conclusions

In the current trend where phytochemicals are gaining interest in the medical and pharmaceutical society. This review summarized and categorized all the studies that were performed on phytochemicals against MM, with the purpose of setting a concise view on the current trend and future perspectives of research. Further reviews targeted on measuring the magnitude and potency of natural product derived drugs should be introduced in the future.

## Figures and Tables

**Table 1:** Intrinsic pathway apoptosis inducing natural products.

Source	Compound	Cell Line	Dose/Duration	Mechanism	References
*Azorella glabra* Wedd. (AG)	AG extract	RPMI8226, SKMM1, MM1S	50 µg/mL; 24, 48 h	c-PARP, c-caspase-3 ↑ Bcl-2 ↓	[34]
*Coptis chinensis* Franch.	Berberine	U266	0, 40, 80, 120, 160 μmol/L; 24 h	PUMA/caspase-3, caspase-9 ↑ Bcl-2 ↓	[35]
*Caesalpinia sappan* (L.) Tod.	Brazilin	U266	60 μM; 0, 6, 12, 24 h	c-caspase-3, c-PARP ↑ Bcl-xL, HDACs ↓	[36,37]
*Boswellia serrata* Spreng.	Boswellic acid	U266	50 μmol/L; 4 h	c-caspase-3, c-PARP ↑ survivin, bcl-xl, bcl-2, Mcl-1 ↓	[38]
*Ligusticum wallichii* Franch.	Tetramethylpyrazine (TMP)	RPMI8226	0, 10, 75, 150, 300 μM; 48 h	c-caspase-3, 8, 9, Bax, Cyto c release, CHOP, cleaved caspase-12, GRP78, GRP94, p-PERK, p-eIF2a, IRE1a, ATF6 ↑ Bcl-2, Bcl-xL ↓	[39]
*Curcuma longa* Linn	Curcumin	U266, RPMI 8226	10 μM; 24 h	c-caspase-3, -8, c-BID, Cyto c release ↑	[40]
*Rheum palmatum* Linn	Emodin	U266, RPMI 8226, IM-9	1, 10, 20, 50, 100 μM/L; 24 h	c-caspase-3, -9 ↑ Mcl-1, JAK2, STAT3 ↓	[41]
*Gardenia jasminoides* J.Ellis	Genipin	U266	100 μM; 0, 24, 48, 72 h	STAT3, c-Src, Bcl-2, Bcl-xL, survivin, cyclin D1, VEGF ↓	[42]
*Panax ginseng* C.A.Mey.	Compound K (CK)	U266	0, 5, 10, 25, 50, 100 μM; 24 h	c-PARP, c-caspase-3 ↑ Bcl-xL, Bcl-2, surviving ↓	[43]
*Sophora flavescens* Aiton	Matrine	U266, RPMI 8226	0.25, 0.5, 1.0, 1.5, 2.0, 3.0 g/L; 48 h	c-caspase-3, cyto c release, Bax ↑ Bcl-2, MMP ↓	[44]
*Rabdosia rubescens* (Hemsl.) H.Hara	Oridonin	U266, RPMI8226	1, 2 μg/mL; 24 h	Mcl-1, Bcl-xL ↓	[45]
*Punica granatum* L.	Pomegrante extract	U266	P. granatum flower extracts: 1, 10, 50, 100 μg/mL; 48, 72 h, P. granatum stem and leaves extracts: 1, 10, 50, 100, 500 μg/mL; 48, 72 h,	MMP ↓	[46]
*Veratrum grandiflorum* Loes	Resveratrol	U266, RPMI 8226	0, 15, 25, 30 μM; 24 h	Bax, c-caspase-3 ↑ cyclin D1, cIAP-2, XIAP, survivin, Bcl-2, Bcl-xL, Bfl-1/A1, TRAF2, AKT ↓	[47]
*Scutellaria baicalensis* Georgi (SB)	SB extract	U266, NCI-H929	50 g/mL; 48 h	p27KIP1, Bax ↑ Bcl-2, Bcl-xL ↓	[48]
*Strychnos nux-vomica* L. (SN)	SN root extract	RPMI 8226	11, 22, 44 mg/mL	cyto C release ↑ MMP ↓	[49]

**Table 2:** Extrinsic pathway apoptosis inducing natural products.

Source	Compound	Cell Line	Dose/Duration	Mechanism	References
*Nigella sativa* Linn	Thymoquinone	MDN, XG-2	10 µM; 24 h	CD95 ↑	[50]

**Table 3:** Intrinsic and Extrinsic pathway apoptosis inducing natural products.

Source	Compound	Cell Line	Dose/Duration	Mechanism	References
*Aplidium albicans*	Alipidin	U266, MM.1S, MM.1R, U266-LR7	0, 1, 2, 5, 10, 20, 50, 100 nmol/L; 72 h	GADD45A, GADD45B, TRAIL, CASP9, CASP6, CIDEC, Smac, c-PARP, c-caspase-3, -7, -8, -9 ↑ MMP, Mcl-1, MMP ↓	[51]
*Blister beetles*	Cantharidin	U266, RPMI 8226, IM-9	5 µM; 24 h	c-caspase -3, -9, c-Bid, Fas ↑ MMP, Bcl-xL ↓	[52]
*Dolabella auricularia*	Dolastatin	U266, RPMI 8226, IM-9	5 nM; 24 h	c-caspase-3, -9, -8, c-Bid, Bax ↑ MMP ↓	[53]
*Camellia sinensis* (L.) Kuntze	EGCG	OPM1	10 µM; 72 h	Fas, Fas ligand, c-caspase -4, p63, DAPK ↑	[54]

**Table 4:** Cell cycle arrest inducing natural products.

Source	Compound	Cell Line	Dose/Duration	Efficacy	References
*Aplidium albicans*	Alipidin	MM.1S, MM.1R	MM.1S: 10 nmol/L; MM.1R: 1 nmol/L	G2/M phase arrest	[51]
*Dolabella auricularia*	Dolastatin	RPMI8226	0–5 nM; 24 h	G2/M phase arrest	[53]
*Tripterygium wilfordii* Hook. f.	Triptolide	RPMI8226	0, 40, 80, 160 nmol/L for 24 h	G2/M phase arrest	[60]
*Caesalpinia sappan* (L.) Tod.	Brazilin	U266	60 μM; 6, 12, 24 h	G2/M phase arrest	[37]
*Boswellia serrata* Roxb. ex Colebr.	Boswellic acid	U266, MM.1S	50 μmol/L;24 h	G2/M phase arrest	[38]
*Punica granatum* L.	Pomegrante extract	U266	100, 250 μg/mL; 24 h	G2/M phase, S phase arrest	[46]

**Table 5:** Angiogenesis inhibiting natural products.

Source	Compound	Cell Line	Dose/Duration	Mechanism	References
*Brucea javanica* (L.) Merr.	Bruceantin (bct)	RPMI 8226 cells, MM-CSC (cancer stem cells)	0, 25, 50, 100 nM; 24 h	Mechanism N/A	[65]
*Scutellaria baicalensis* Georgi	Wogonin	U266 RPMI 8226	(in vitro): 20, 40, 80 μM; 24 h (in vivo): 0, 40, 80 mg/kg (i.v. injection); 24 h	<in vitro> VEGF, c-Myc, HIF-1α ↓ <in vivo> c-Myc, HIF-1a, VHL, VEGF ↓	[66]
*Citrus paradise* Macfad.	Bergamottin	U266	100 μM; 0, 6, 12, 24 h	COD-X, VEGF, cyclin D1, IAP-1, Bcl-2, Bcl-xL ↓	[67]
*Solenopsis invicta* Buren	Solenopsin A	(in vitro) SVR cell proliferation (in vivo) zebrafish model system	(in vitro) 0, 1, 3, 6 µg/mL; 48 h (in vivo) 6 µg/mL; duration N/A	Akt↓, FOXO1a ↓	[68]
*Carpobrotus edulis* (L.) N.E.Br.	Rutin–Zinc (II) Flavonoid–Metal Complex	RPMI8226	17.2–275.6 μM; 24 h	Caspase-3, Caspase-8 ↑ VEGF, cyclin D1 ↓	[69]
*Artemisia annua* Linn.	Artesunate	RPMI8226	3, 6, and 12 μmol/L; 48 h	Mechanism N/A	[70]

**Table 6:** miRNA regulating natural products.

Source	Compound	Cell Line	Dose/Duration	Mechanism	References
*Coptis chinensis* Franch	Berberine	U226, RPMI 8266	40, 80, 120, 160 μmol/L 24, 48, 72 h	miR-21, miR-17-92, miR-99a-125b, miR-106-25 ↓	[35,89,90]
*Tripterygium wilfordii* Hook. F (TWHF)	Triptolide	MM.1S	2.5–40 ng/mL; 24 h	miR142-5p/miR181a ↓	[91]

**Table 7:** Clinical trials of natural products about MM.

Source	Compound	Phase	Patients	Status	Nct Number	References
*Agaricus blazei* Murrill	*Agaricus blazei* extract	II	33	completed	NCT00970021	[92,93,94]
*Curcuma longa* Linn	Curcumin (Diferuloylmethane derivative)	pilot study	33	completed	NCT00113841	[40]
shark cartilage	Neovastat (AE-941)	II	125	completed	NCT00022282	[95]

## Abbreviations

MM	Multiple myeloma
IMiDs	immunomodulatory drugs
PIs	proteasome inhibitors
IMWG	International Multiple Myeloma Working Group
CRS	cytokine release syndrome
FADD	Fas-associated death domain
ROS	Reactive oxygen species
HDACs	histone deacetylases
HATs	histone acetyltransferases
TMP	tetramethylpyrazine
CK	Compound K
SB	*Scutellaria baicalensis*
TQ	Thymoquinone
EGCG	Epigallocatechin-3-gallate
DAPK2	death-associated protein kinase 2
VEGF	vascular endothelial growth factor
PDGF	platelet-derived growth factor
FGF	fibroblast growth factor
EGF	epidermal growth factor
TGFβ	transforming growth factor beta
MMPs	matrix metalloproteinase’s
TNF	tumor necrosis factor
Ang-1	angiopoietins
uPAR	urokinase receptor
BM	bone marrow
BMM	bone marrow microenvironment
PCDC4	programmed cell death 4
GR	glucocorticoid receptors
ABM	*Agaricus blazei* Murrill
PBMCs	peripheral blood mononuclear cells
